# The control of viral infection by tripartite motif proteins and cyclophilin A

**DOI:** 10.1186/1742-4690-4-40

**Published:** 2007-06-12

**Authors:** Greg J Towers

**Affiliations:** 1 MRC Centre for Medical Molecular Virology, Department of Infection, Royal Free and University College London Medical School, 46 Cleveland Street, London, W1T4JF, UK

## Abstract

The control of retroviral infection by antiviral factors referred to as restriction factors has become an exciting area in infectious disease research. TRIM5α has emerged as an important restriction factor impacting on retroviral replication including HIV-1 replication in primates. TRIM5α has a tripartite motif comprising RING, B-Box and coiled coil domains. The antiviral α splice variant additionally encodes a B30.2 domain which is recruited to incoming viral cores and determines antiviral specificity. TRIM5 is ubiquitinylated and rapidly turned over by the proteasome in a RING dependent way. Protecting restricted virus from degradation, by inhibiting the proteasome, rescues DNA synthesis, but not infectivity, indicating that restriction of infectivity by TRIM5α does not depend on the proteasome but the early block to DNA synthesis is likely to be mediated by rapid degradation of the restricted cores. The peptidyl prolyl isomerase enzyme cyclophilin A isomerises a peptide bond on the surface of the HIV-1 capsid and impacts on sensitivity to restriction by TRIM5α from Old World monkeys. This suggests that TRIM5α from Old World monkeys might have a preference for a particular capsid isomer and suggests a role for cyclophilin A in innate immunity in general. Whether there are more human antiviral TRIMs remains uncertain although the evidence for TRIM19's (PML) antiviral properties continues to grow. A TRIM5-like molecule with broad antiviral activity in cattle suggests that TRIM mediated innate immunity might be common in mammals. Certainly the continued study of restriction of viral infectivity by antiviral host factors will remain of interest to a broad audience and impact on a variety of areas including development of animal models for infection, development of viral vectors for gene therapy and the search for novel antiviral drug targets.

## Background

The control of viral infection by intracellular antiviral proteins referred to as restriction factors has become an important and challenging focus of infectious disease research. A clearer understanding of the role of restriction factors in immunity and the control of retroviral replication promises to reveal details of host virus relationships, allow improvement of animal models of infection, identify targets for antiviral therapies, and further facilitate the use of viral vectors for clinical and investigative gene delivery. The tripartite motif protein TRIM5α has recently emerged as an important restriction factor in mammals blocking infection by retroviruses in a species-specific way. Early evidence for TRIM5α 's antiviral activity included the species-specific infectivity of retroviral vectors, even when specific envelope/receptor requirements were obviated by the use of the VSV-G envelope. Notable examples include the poor infectivity of certain murine leukemia viruses (MLV) on cells from humans and primates and the poor infectivity of HIV-1 on cells from Old World monkeys [1-3]. The notion that a dominant antiviral factor was responsible was suggested by the demonstration that the block to infection could be saturated, or abrogated, by high doses of retroviral cores [4-6]. The putative human antiviral factor was named Ref1 and the simian factor Lv1 [1,6]. TRIM5α was identified in 2004 by screening rhesus cDNAs for those with antiviral activity against HIV-1 [7]. Shortly after, several groups demonstrated that Ref1 and Lv1 were encoded by species-specific variants of TRIM5α [8-11]. TRIM5α therefore represents a hitherto undescribed arm of the innate immune system, blocking infection by an incompletely characterised mechanism. Its expression is induced by interferon via an IRF3 site in the TRIM5 promoter linking it to the classical innate immune system [12].

### The tripartite motif

TRIM5 has a tripartite motif, also known as an RBCC domain, comprising a RING domain, a B Box 2 domain and a coiled coil [13,14]. The RING is a zinc-binding domain, typically involved in specific protein-protein interactions. Many RING domains have E3 ubiquitin ligase activity and TRIM5 can mediate RING dependent auto-ubiquitinylation *in vitro *[15]. B boxes are of 2 types, either B-box1 or B-box2 and TRIM5 encodes a B-box2. B-boxes have a zinc-binding motif and are putatively involved in protein-protein interactions. The two types of B-box have distinct primary sequence but similar tertiary structures and are structurally similar to the RING domain. This suggests that they may have evolved from a common ancestral fold, and perhaps have a similar function, such as ubiquitin ligation [16,17]. It is also possible that B-Boxes contributes to ligation specificity, ie have E4 activity [16,17]. The coiled-coil is involved in homo- and hetero-multimerisation of TRIM proteins [14,18]. TRIM5 exists as a trimer with the coiled coil facilitating homo and hetero multimerisation with related TRIM proteins [18-20].

TRIM5 RNA is multiply spliced, generating a family of isoforms, each shorter from the C terminus. The longest, TRIM5α, encodes a C terminal B30.2 domain that interacts directly with viral capsid and determines antiviral specificity [18,21,22]. The shorter isoforms, TRIM5γ and TRIM5δ, do not have B30.2 domains and act as dominant negatives to TRIM5α and rescue restricted infectivity when over-expressed [7,23]. It is assumed that the shorter forms form heteromultimers via the coiled coil and titrate the viral binding B30.2 domains. It is therefore possible that TRIM5's antiviral activity is regulated by splicing.

The B30.2 domain comprises a combination of a PRY motif followed by a SPRY motif [24]. Whilst SPRY domains are evolutionary ancient, B30.2 domains, found in butyrophilin and TRIM proteins, appeared more recently. There is unlikely to be a precise function for B30.2 domains, rather they are involved in protein-protein interactions such as substrate recognition. A series of TRIM5 mutagenesis studies demonstrated that the TRIM5 B30.2 domain determines antiviral specificity and defined the specific regions of the B30.2 responsible [18,21,22,25,26]. In vitro capsid/TRIM5 binding assays have been developed and these demonstrate that, at least in the case of wild type TRIM5α proteins, binding correlates well with the ability to restrict infection [27,28].

The recent solution of the structure of several B30.2 domains allows us to interpret the conservation and variation between TRIM5 B30.2 domains [29-31]. The structures indicate that the B30.2 core is formed from a distorted 2-layer beta sandwich with the beta strands in an anti-parallel arrangement. Extending from the core are a series of loops and it is these surface loop structures that vary between the TRIM5 sequences from each primate and between different B30.2 domains of TRIM5 homologues. The loops form 3 or 4 variable regions, all of which appear to impact on antiviral specificity [32]. The TRIM21 structure in complex with its ligand, IgG Fc indicates that there are 2 binding surfaces, one in the PRY (V1) and 1 in the SPRY (V2-V3) and this is likely to be true for TRIM5α.

### TRIM5 and the Red Queen

B30.2 mutagenesis studies, as well as sequence analysis of TRIM5α from related primates, suggested that the differences defining anti-viral specificity are concentrated in patches in the B30.2 domain [33]. The patches, which correspond to the surface loops, have been under very strong positive selection as evidenced by a high dN:dS ratio. dN:dS ratios have been calculated by comparing TRIM5 sequences from primates and comparing the number of differences that lead to a change in the protein sequence (non synonomous, dN) to the number of differences that do not (synonomous, dS). A high ratio indicates positive selection and is evidence of the host-pathogen arms race known as the Red Queen hypothesis [34]. This phenomenon, named after Lewis Carroll's Red Queen who claimed 'It takes all the running you can do to keep in the same place', refers to the selection driven genetic change that occurs in both host and pathogen as each alternately gains the advantage. Whether selection pressure on TRIM5 has been from pathogenic retroviruses or from endogenous retroviruses and retrotransposons is unclear. The relative youth of lentiviruses, as compared to other retroviruses and endogenous elements, is thought to preclude them from impacting on TRIM5 selection, although the discovery of an endogenous lentivirus in rabbits [35] has recently extended their age from less than 1 million years to greater than 7 million years and it certainly seems possible that this age will extend further as we better understand lentiviral history.

The other side of the Red Queen's arms race is the change in the retroviral capsids to escape restriction by TRIM5. TRIM5 molecules can generally restrict widely divergent retroviruses including gamma retroviruses as well as lentiviruses. For example Agm and bovine TRIMs restrict MLV-N, HIV-1, HIV-2 and SIVmac [36-38]. It is now clear that retroviral capsid structures are conserved and capsid hexamers are found in both lentiviruses and gamma retroviruses [39,40] so we imagine that the TRIMs recognise a conserved shape. Paradoxically, point mutants can often escape strong restriction. MLV-N CA R110E escapes human, simian and bovine TRIMs, SIVmac CA QQ89-90LPA escapes rhesus and squirrel monkey TRIM5s and HIV-1 G89V escapes owl monkey TRIMCyp [1,38,41-44]. It therefore remains unclear how TRIM5 can be effective if a small number of changes in CA can rescue infectivity, especially given that retroviral capsid sequences appear quite plastic.

### The antiviral mechanism

**Figure 1:**
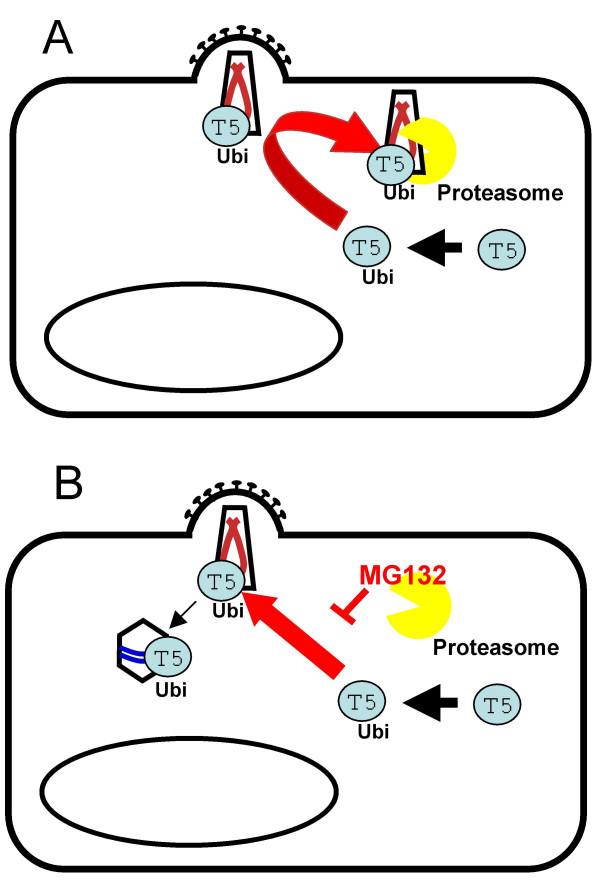
**A putative mechanism for restriction of retroviruses by TRIM5α**. (Panel A) TRIM5α is autoubiquitinylated in a RING dependent way and rapidly turned over by the proteasome [47]. If it encounters incoming sensitive retroviral cores then they too are recruited to the proteasome and destroyed, before the virus has the opportunity for significant reverse transcription. (Panel B) If the virus/TRIM5α complex is protected from destruction, by inhibiting the proteasome, then the virus can reverse transcribe [48, 49]. Infectivity is not rescued however, indicating that the virus/TRIM5α complex is uninfectious. How TRIM5 renders the virus uninfectious remains unclear.

We are beginning to understand TRIM5α 's antiviral mechanism. TRIM5α is trimeric [19,45] and interacts with hexameric capsids [46]. TRIM5α is ubiquitinylated within cells and is rapidly turned over by the proteasome in a RING domain dependent way suggesting that autoubiquitinylation might drive this process [15,47]. We imagine that the rapid turnover of TRIM5α and presumably TRIM5α-virus complexes leads to an early block to infection, before the virus has had the opportunity to reverse transcribe (Figure 1A). This notion is supported by the observation that inhibition of the proteasome during restricted infection allows the virus to reverse transcribe, when it is protected from degradation [48,49] (Figure 1B). However, infection is not rescued by inhibition of the proteasome, indicating that the TRIMα-virus complex remains uninfectious, even when protected from degradation. How exactly TRIM5α renders the virus uninfectious remains unclear, but it may be that by simply coating the core with multivalent complexes TRIM5α trimers are able to disrupt the rearrangement/uncoating and or trafficking required to continue to the nucleus and to integrate. Other possibilities include TRIM5α rapidly uncoating incoming HIV-1 capsids. In fact, this has been observed using an assay of capsid density to measure uncoating [46,50] and it will be interesting perform this assay in the presence and absence of proteasome inhibitors to address whether the proteasome has a role this process. Proteasome independent degradation of capsids by TRIM5α has also been described [51]. Importantly, DNA circles remain inhibited, even in the absence of proteasome activity, suggesting that the restricted TRIM5α-virus complex cannot access the nucleus. [48,49] (Fig 1). It is possible that these observations indicate several independent antiviral activities of TRIM5α but we prefer the interpretation that there are several possible fates for a restricted virion. It may be degraded by the proteasome, it may inappropriately uncoat, or it may remain intact, make DNA but not have access to the nucleus. The different fates are likely to be influenced by factors such as the particular virus, the particular TRIM5α as well as virus dose and TRIM5α expression levels and the cellular background. Understanding the contribution of these activities to restriction by TRIM5α will require further study but the field continues to make steady progress.

### A Role for Cyclophilin A in restriction

The relationship between Cyclophilin A (CypA) and HIV-1 has a long history. CypA is a peptidyl prolyl isomerase that performs cis/trans isomerisation of proline peptide bonds in sensitive proteins. CypA interacts with gag in infected cells leading to its recruitment into nascent HIV-1 virions [52,53]. Recent data has shown that CypA also interacts with incoming HIV-1 cores in newly infected cells and that this interaction is more important for infectivity than that occurring as cores assemble [42,54-56]. This may be because only about 10% of the capsid molecules in the core recruit a CypA molecule into the virion [52,53]. CypA performs cis/trans isomerisation at CA G89-P90 on the outer surface of the capsid [57,58] and this leads to changes in infectivity. In Old World monkey (OWM) cells CypA decreases HIV-1 infectivity, but only in the presence of TRIM5α [59-61]. Blocking CypA activity using the immunosuppressant competitive inhibitor of CypA cyclosporine A (CSA), or reducing CypA expression with small interfering RNA, reduces the susceptibility of HIV-1 to restriction by OWM TRIM5 and rescues HIV-1 infectivity.

In human cells the interaction between incoming HIV-1 cores and CypA is important for maximal infectivity. Preventing this interaction reduces HIV-1 infectivity independently of TRIM5 expression [59,62]. It is suspected that in the absence of CypA activity, HIV-1 gets restricted by a TRIM5 independent antiviral activity. This suspicion is borne from the fact that the requirement for CypA is both cell type, and species, specific, suggesting that CypA is not required simply to uncoat the core. This notion is further supported by the observation that CA point mutants close to the CypA binding site such as HIV-1 CA A92E or G94D appears to lead to restriction of HIV-1 in human cells [55,56]. A92E or G94D infectivity is reduced in some human cell lines but not others and strikingly, infectivity is rescued by inhibition of CypA. It is possible that these mutants become sensitive to human restriction factor(s) and that the interaction between the factor and the virion is sensitive to the activity of CypA on the peptide bond at P90.

**Figure 2:**
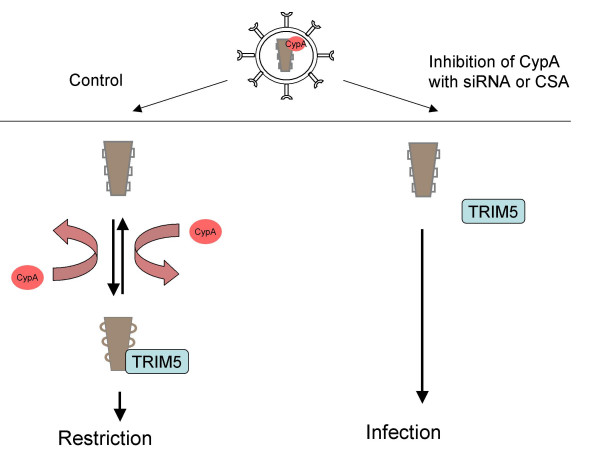
**A putative mechanism for activity of CypA on HIV-1 infectivity in cells from Old World monkeys**. HIV-1 recruits CypA to around 10% of its capsid monomers in newly assembled cores [52, 53]. When the core enters the cytoplasm of a target cell it recruits more CypA, which efficiently catalyses cis/trans isomerisation of the peptide bond at CA G89-P90 [42, 57]. This activity replenishes the cis conformation CA as it is recruited into the restricted complex with TRIM5α. If CypA activity is reduced in target cells, using CypA specific siRNA or by inhibiting CypA activity with CSA, then the OWM TRIM5α cannot interact with the CA, which is mostly in the trans conformation, and infectivity is rescued [59-61]. The isomerisation at CA G89-P90 is represented by squares (trans) changing to circles (cis) on the surface of the capsid.

How might CypA impact on recognition of CA by TRIM5α? One possibility is that in some cases, capsid with CypA attached may make a better target for TRIM5α. This possibilty has been discounted on the basis that HIV-1 mutated to prevent CypA binding (HIV-1 CA G89V) remains restricted by TRIM5α from Old World monkeys [59,61]. Importantly, this mutant is not restricted by TRIM-Cyp, which relies on the CypA domain to recruit it to the HIV-1 capsid [43]. A second possibility is that recruitment of TRIM5α to capsid is improved by the prolyl isomerisation activity of CypA on HIV-1 capsid. Prolyl isomerisation has been shown to regulate protein-protein interaction in diverse biological systems including the control of cell division by cdc25C and signalling by the Itk receptor. The prolyl isomerase Pin1 catalyses the cis/trans isomerisation of a proline peptide bond in cdc25C. Cdc25C activity is regulated by phosphorylation and since its phosphatase PP2A only recognises the cdc25C trans isomer, Pin1 activity leads to dephosphorylation and cdc25C activation [63]. A similar molecular switch has been described for Itk signalling and CypA. CypA catalyses cis/trans isomerisation of proline 287 in the Itk SH2 domain impacting on interaction with phosphorylated signalling partners and regulating Itk activity [64,65]. NMR measurements have shown that HIV-1 CA contains around 86% trans and 14% cis at G89-P90 in both the presence and absence of CypA [57]. However, in the presence of CypA, CA is rapidly isomerised between the two states [57]. It is therefore possible that OWM TRIM5α binds preferentially to CA containing G89-P90 in the cis conformation [59]. In this case, in the presence of TRIM5α, CypA maintains the percentage of cis at 14% even as TRIM5α sequesters it from the equilibrium. In this way the trans form is isomerised to cis and becomes bound by TRIM5α. Blocking CypA activity would limit the availability of the cis conformation and therefore TRIM5α's ability to see the CA, resulting in rescued infectivity. This model is summarised in Fig 2. CypA also appears to impact on replication of feline immunodeficiency virus in feline and human cells although whether TRIM5 is required for this remains unclear [66].

Surprisingly in the New World species owl monkey a CypA pseudogene has been inserted into the TRIM5 coding region, replacing the viral binding B30.2 domain with CypA, leading to a molecule called TRIMCyp [43,44]. This restriction factor strongly restricts HIV-1, SIVagm and FIV by recruitment of the incoming capsid to the RBCC domain facilitated by interaction between the CypA domain and the capsid [20,66,67]. Viral infectivity is rescued by inhibition of CypA-CA interactions with CSA indicating the dependence on CypA binding to capsid for robust restriction. We assume that at some point in owl monkey evolution the modification of TRIM5 to TRIMCyp provided a significant selective advantage. We can only speculate on what might have provided the selection pressure but a pathogenic virus that recruited CypA is a possibility. It is worth noting that a TRIMCyp in the human genome would be a useful antiviral as we face the current AIDS pandemic.

**Figure 3:**
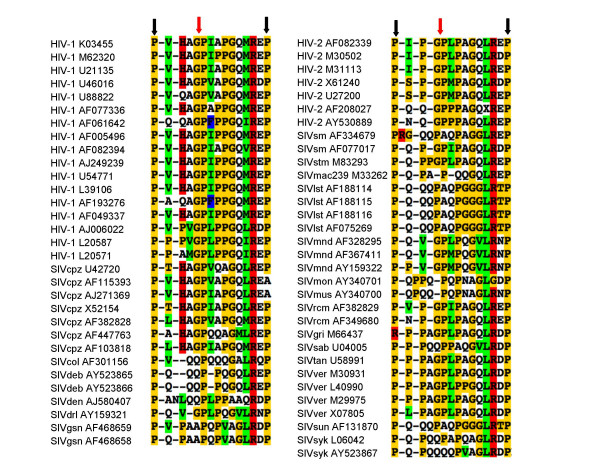
**Similarity between the sequences of retroviral capsids**. Alignment of primate lentiviral capsid protein sequences demonstrates that they have conserved the proline rich Cyclophilin A binding loop on their outer surface. Glycine proline motifs are common (red arrow). Conserved prolines at the extremes of the loop are shown (black arrows). The alignment from which this selection was taken is available from the Los Alamos HIV sequences database [93]. Retroviruses are named according to the species from which they were isolated. Genbank accession numbers are shown. Species abbreviations are as follows: cpz chimpanzee, deb De Brazza's monkey, den Dent's Mona monkey, drl drill, gsn greater spot nosed monkey, sm sooty mangabey, stm stump tailed macaque, mac rhesus macaque, lst L'Hoest monkey, mnd mandrill, mon Cercopithecus mona, mus Cercopithecus cephus, rcm red capped mangabey, gri African green monkey Grivet, sab African green monkey sabaeus, tan African green monkey tantalus, ver African green monkey vervet, sun sun tailed monkey, syk Sykes monkey.

**Figure 4:**
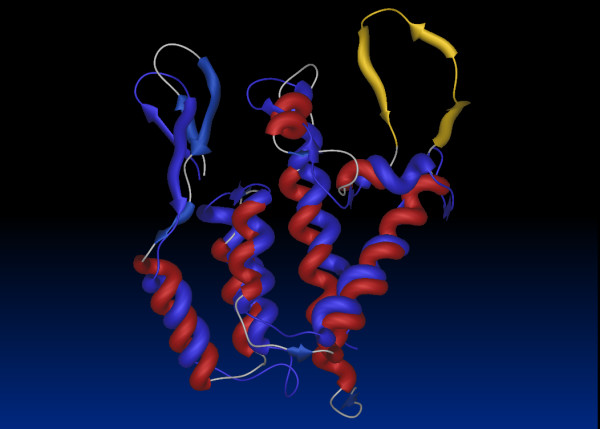
**Similarity between the structures of retroviral capsids**. Superimposition of the structures of the N terminal domains of HIV-1 (Red) and MLV (blue) capsids demonstrates overall structural conservation although the Cyclophilin A binding loop (yellow) is absent in MLV. The pdb files for HIV-1 (1M9C) [94] and MLV (1UK7) [40] were superimposed using pairwise structure comparison [95].

The role of CypA in sensitivity to TRIM5, its fusion to TRIM5 in owl monkeys and its role as a target for immunosuppression implies that CypA might have a general role in immunity. Viruses are likely to be under considerable pressure to alter their shape and become invisible to antiviral shape recognition systems such as TRIMs. Molecules, such as CypA, that induce shape changing, may have an important role in making escape difficult. For example, HIV-1 appears to be invisible to OWM TRIM5 in the absence of CypA, but in its presence HIV-1 is strongly restricted [59-61]. Conversely, HIV-1 is highly infectious in human cells in the presence of CypA but appears to become restricted in its absence [42]. It seems that HIV-1 is invisible to human TRIM5 whether CypA is active or not but becomes restricted by something else in the absence of CypA activity [59,62]. HIV-1 appears to have adapted to tolerate CypA activity and this adaptation has made it dependent on CypA. Why can't HIV-1 simply avoid recruiting CypA? The answer to that is not clear but a clue can be found in alignment of the CypA binding region of lentiviruses (Figure 3). All primate lentiviruses have conserved the proline rich CypA binding loop and many encode glycine proline motifs within it. This suggests that the motifs that recruit CypA are important, conserved and cannot easily be mutated. The loops and glycine proline motifs are also conserved in the equine lentivirus EIAV and the feline FIV [67]. Their purpose however remains unclear and this loop is not conserved in MLV [40] (Figure 4).

### Polymorphism and TRIM5 in other species

The fact that TRIM5 restricts retroviral infection so potently, at least in monkeys, has suggested that polymorphism in human TRIM5 might impact on HIV-1 transmission and/or pathogenesis *in vivo*. Several studies have addressed this issue and shown at best, only weak association of any particular TRIM5α allele with disease progression [68-71]. Importantly, human TRIM5α is not polymorphic in the regions of the B30.2 domain known to impact on viral recognition, and its over expression does not reduce HIV-1 infectivity by more than a few fold [7,9,10,72]. Furthermore under *in vitro *conditions where rhesus TRIM5 efficiently binds the HIV-1 capsid, the human protein binds only poorly [46]. It therefore seems likely that TRIM5 doesn't significantly impact on HIV-1 replication and pathogenesis in humans. Indeed, we imagine that HIV-1's insensitivity to TRIM5 has been an important factor in its success as a pathogen in humans. Conversely the TRIM5 gene in rhesus macaques and sooty mangabeys is relatively polymorphic with a number of polymorphisms occurring in the variable loops that dictate antiviral specificity. Indeed, expression of these alleles in permissive feline cells followed by challenge with retroviral vectors derived from HIV-1, SIVmac MPMV or MLV-N demonstrated that the different alleles have slightly different antiviral specificities [72].

The antiviral activity of TRIMs in mammals other than primates remains less well characterised. A bovine TRIM (BoLv1) with broad anti retroviral activity suggests that TRIM-mediated restriction of retroviruses is widespread amongst mammals [37,38]. BoLv1 is closely related to primate TRIM5 genes suggesting that they are orthologs derived from an ancestral antiviral TRIM. Cattle encode at least 4 genes closely related to TRIM5, in addition to homologs of TRIM34 and TRIM6. The fact that one of these proteins has antiviral activity supports the notion that these genes are derived from an ancestral sequence with antiviral activity. It is likely that antiviral TRIMs will be identified in more mammals soon. Indeed, antiviral TRIMs are probably responsible for the poor infectivity of cells from pigs and bats to MLV-N and those of rabbits to HIV-1 [1,3,5,73].

### Are there other TRIMs with antiviral properties?

Protein families arise through the duplication of ancestral gene sequences and therefore members of a family share common ancestry. Human TRIM5 lies on chromosome 11 within a group of closely related TRIMs, comprising TRIMs 5, 6, 34 and 22, which have presumably arisen by gene duplication. These TRIMs, as well as TRIMs 1, 18, 19 and 21 have no, or relatively weak, antiviral activity against a panel of distantly related retroviruses including HIV-1, HIV-2, SIVmac, EIAV and MLV [20]. Whether this is because they have an alternate function or whether they are simply not active against this selection of viruses is difficult to say. It is worth noting however that comparison of the sequences of these TRIMs from primates shows that unlike TRIM5, TRIMs 6, 22 and 34 do not have strongly selected B30.2 domains, suggesting that they have not been under the same selection pressures as TRIM5 [74].

There is an increasing body of evidence, gathered over many years suggesting that TRIM19, otherwise known as PML, may have antiviral activity. PML exists in sub-nuclear structures called PODs, ND10 or PML bodies and are of unclear function. It has long been known that a number of diverse viruses including influenza, SV40 and papilloma virus form replication complexes in close association with PML bodies, reviewed in [75,76]. Infection by other viruses, including herpes viruses and adenoviruses, causes degradation of PML protein and dispersal of the body components. The molecular details of PML degradation by herpes simplex type 1 (HSV-1) have been partially solved. The HSV-1 protein ICP0 is responsible for inducing proteasome dependent degradation of PML, and HSV-1 deleted for this protein replicates poorly, leaving PML bodies intact [77-80]. Importantly, mutant HSV-1 (ICP0-) becomes almost fully infectious if PML expression is reduced using RNA interference, indicating that an important function of ICP0 is to eliminate PML [81]. An antiviral role for PML is also suggested by a real time microscopy study demonstrating that PML is recruited to incoming HSV-1 (ICP0-) replication complexes [82]. Such active recruitment is strongly suggestive of an antiviral response. Furthermore, reduction of PML expression increases permissivity of human cells to human cytomegalovirus infection [83], and over-expression of PML reduces permissivity to vesicular stomatitis virus and influenza A [84,85]. These data, along with the observation that PML expression is stimulated by type 1 interferon, strongly support an antiviral role for TRIM19 (PML). Interestingly, PML does not have a B30.2 domain suggesting that it interacts with target viruses in a different way to TRIM5α interacting with retroviruses.

Further data supporting an antiviral role for TRIM proteins comes from expression studies in which TRIMs are expressed in permissive cells and the modified cells tested for permissivity to infection by retroviral vectors. Such studies have demonstrated weak anti-retroviral activity of TRIM1 from African green monkeys and Owl monkeys against MLV-N [9]. It is also worth noting that a particular TRIM protein can impact on viral infectivity by influencing the activity of another antiviral TRIM protein. For example, expression of TRIM34 can reduce the antiviral activity of TRIM5 presumably via heteromutimerisation mediated via the coiled coil [20]. This observation suggests a complex mechanism of regulation and generation of alternate antiviral specificities through heteromultimerisation. Whether further TRIMs have antiviral activity remains largely untested. The fact that TRIMs 10, 15, 26, 27, 31, 38, 39, 40 are associated with the major histocompatibility complex on chromosome 6 [86] and the observation that the expression of most of these genes is up-regulated by influenza infection [87] suggests that they might have a role in immunity.

TRIM20, otherwise known as pyrin, presents as an intriguing antiviral possibility. Polymorphism in the TRIM20 B30.2 domain can cause familial Mediterranean fever, a disease characterised by recurrent attacks of fever and inflammation. Sequencing TRIM20 from a variety of primates revealed that many encode the disease causing mutations as wild type sequence [88]. Furthermore, phylogenetic analysis suggested episodic selection in the B30.2 domain, similar to that seen for TRIM5, suggesting the intriguing possibility that viral infection underlies this disease. Rather strikingly in 2001 these authors suggested that the B30.2 domain of pyrin might interact directly with pathogens and that the mutations are counter evolutionary changes selected to cope with a changing pathogen [88]. Such a model is remarkably close to what we believe to be true for TRIM5, retroviruses and the Red Queen 6 years later.

### Concluding Remarks

Just as we considered that the important aspects of TRIM5 biology had been largely described, the Ikeda lab described tantalising findings that make a complicated subject significantly more complicated [89]. They show that rhesus TRIM5 causes degradation of gag in infected cells. Importantly this activity is independent of the C-terminal B30.2 domain suggesting that it acts via an alternative specificity determinant, perhaps the coiled coil. It is worth noting that APOBEC3G has also been described as being able to restrict infection of both incoming as well as outgoing HIV-1 [90,91]. It may be therefore that such dually active restriction factors are not uncommon.

Whether the study of host factors influencing viral infection will translate into improvements in antiviral therapy in the foreseeable future remains uncertain. However, it is likely to allow the improvement of animal models for HIV/AIDS as we enhance our understanding of the viral and cellular determinants for viral replication and disease [92]. This work is also likely to improve our ability to transduce cells, therapeutically and experimentally, with viral gene delivery vectors, particularly poorly permissive primary cells and stem cells. It certainly promises to remain an active and exciting field in infectious disease research.

## Abbreviations

TRIM, tripartite motif; MLV, murine leukemia virus; MLV-N, N tropic MLV; MLV-B, B tropic MLV; CypA Cyclophilin A; CSA, cyclosporine A; CA, capsid

## Competing interests

The author(s) declare that they have no competing interests.
